# Neuroimage Biomarker Identification of the Conversion of Mild Cognitive Impairment to Alzheimer’s Disease

**DOI:** 10.3389/fnins.2021.584641

**Published:** 2021-02-19

**Authors:** Te-Han Kung, Tzu-Cheng Chao, Yi-Ru Xie, Ming-Chyi Pai, Yu-Min Kuo, Gwo Giun Chris Lee

**Affiliations:** ^1^MediaTek Inc., Hsinchu, Taiwan; ^2^Department of Electrical Engineering, National Cheng Kung University, Tainan, Taiwan; ^3^Department of Radiology, Mayo Clinic, Rochester, MN, United States; ^4^Division of Behavioral Neurology, Department of Neurology, College of Medicine, National Cheng Kung University Hospital, National Cheng Kung University, Tainan, Taiwan; ^5^Alzheimer’s Disease Research Center, National Cheng Kung University Hospital, Tainan, Taiwan; ^6^Institute of Gerontology, College of Medicine, National Cheng Kung University, Tainan, Taiwan; ^7^Department of Cell Biology and Anatomy, College of Medicine, National Cheng Kung University, Tainan, Taiwan

**Keywords:** mild cognitive impairment, Alzheimer’s disease, magnetic resonance imaging, hippocampal subfields, multilayer perceptron

## Abstract

An efficient method to identify whether mild cognitive impairment (MCI) has progressed to Alzheimer’s disease (AD) will be beneficial to patient care. Previous studies have shown that magnetic resonance imaging (MRI) has enabled the assessment of AD progression based on imaging findings. The present work aimed to establish an algorithm based on three features, namely, volume, surface area, and surface curvature within the hippocampal subfields, to model variations, including atrophy and structural changes to the cortical surface. In this study, a new biomarker, the ratio of principal curvatures (RPC), was proposed to characterize the folding patterns of the cortical gyrus and sulcus. Along with volumes and surface areas, these morphological features associated with the hippocampal subfields were assessed in terms of their sensitivity to the changes in cognitive capacity by two different feature selection methods. Either the extracted features were statistically significantly different, or the features were selected through a random forest model. The identified subfields and their structural indices that are sensitive to the changes characteristic of the progression from MCI to AD were further assessed with a multilayer perceptron classifier to help facilitate the diagnosis. The accuracy of the classification based on the proposed method to distinguish whether a MCI patient enters the AD stage amounted to 79.95%, solely using the information from the features selected by a logical feature selection method.

## Introduction

Alzheimer’s disease (AD) is the most common form of dementia, representing a significant burden on the global economy (Prince et al., 2015). While the treatment of AD remains a major clinical challenge, slowing down the deterioration of cognitive capability during the mild cognitive impairment (MCI) stage represents an important preventive approach. Consequently, it is critical to monitor whether a patient is progressing from MCI to AD (a converter) or is still in the MCI stage (a non-converter).

Multiple AD biomarkers have been recognized with varying trends as the disease progresses (Bateman et al., 2012). For the prediction of AD, amyloid, tau, and neurodegeneration are related and efficient biomarkers that comply with the amyloid hypothesis. This hypothesis postulated that AD is due to a cascade mechanism, starting from the deposition of Aβ and tau hyperphosphorylation, which then further causes neurodegeneration, including synaptic dysfunction, death of neural cells, and brain shrinkage (Hardy and Selkoe, 2002; Spillantini and Goedert, 2013). However, the examinations of amyloid and tau, including the extraction of cerebrospinal fluid (CSF) and contrast injection for a PET scan, are in general more invasive. Therefore, we resort to the last potential mechanism in studying AD—neurodegeneration. Neurodegeneration encompasses structural variations, such as atrophy and neuronal loss due to amyloid and tau deposition, which can be measured and, hence, quantified *via* structural MRI. Clear observations of the hippocampal volume using this neuroimaging modality are indicative biomarkers revealing the obvious changes in the transition from MCI to AD which have been well documented (Frisoni et al., 2010; Bateman et al., 2012).

Studies on structural atrophy in the hippocampus and entorhinal cortex, based on the evaluation of volume and surface area from MRI scans during the progression from MCI to AD, have been previously carried out (Devanand et al., 2007; Frisoni et al., 2010; Bateman et al., 2012). Although the volume and surface area of the hippocampus and entorhinal cortex have been observed to be highly correlated to AD (Bobinski et al., 2000; Dickerson et al., 2009), these biomarkers are influenced by natural aging (Bigler et al., 1997; Dickerson et al., 2009). The curvature of the cortical surface is another feature in the analysis of cognitive evolution. It has been shown that the mean curvature of the cortex is less influenced by normal aging (Long et al., 2012). The mean curvature derived from a surface analysis could be used to distinguish MCI from AD based on the analysis of the cortical area (Long et al., 2013).

While some AD biomarkers have achieved good prediction, most of them require several different examined data modalities to achieve good accuracy. In this work, we propose a method to screen for whether an MCI patient has developed AD, using only the MRI data, with efficiency and good accuracy. This work analyzes geometric features to identify the conversion from MCI to AD by characterizing structural changes in the hippocampus. For surface curvature, we further introduce a new feature, the “average of principal curvature ratio,” instead of using the average curvature. In addition to the statistical evaluation of indices, including the volume, surface area, and curvature index within the hippocampus, the multilayer perceptron (MLP) classifier is designed to predict whether a subject has converted from MCI to AD.

### Recent Advances

Recently, internationally encoded endpoints, e.g., clinical, imaging, genetic, and biospecimen biomarkers, have been introduced together with machine learning analytics algorithms in predicting and characterizing the disease process from normal aging to early MCI, late MCI, and dementia, especially AD. Neuroimaging biomarkers have especially gained popularity in providing direct indication of the AD progress.

A systematic review primarily on imaging and biochemical biomarkers, including primarily MRI, PET scans, and CSF or plasma amyloid-β/tau, with longitudinal cohorts in anticipating characterization of AD progression has been documented (Lawrence et al., 2017). Principal component analysis was used by Blazhenets et al. (2019) to quantify cerebral metabolic patterns measured from fluorodeoxyglucose-positron emission tomography (FDG-PET) related to MCI to AD conversion. Clinical variables were also used. FDG-PET brain images were used by Brown et al. (2020) at different prodromal stages in tracking longitudinally the AD process. Statistical textural features on the entorhinal cortex from MRI scans were extracted for differentiation of normal control, MCI, and AD by Leandrou et al. (2020). Textural biomarkers were assessed for its superiority over traditional volumetric features in earlier indication of brain atrophy. Similarly, Lee et al. (2020) also extracted grayscale co-occurrence matrix texture features surrogating as hippocampus precuneus and posterior cingulate cortex biomarkers. Structural MRI (sMRI) cortical and subcortical measurements, e.g., thickness and rs-fMRI functional graph connectivity biomarkers, were studied with SVM classification having high prediction accuracy for MCI converter or non-converter (Cabral et al., 2015). Vuoksimaa et al. (2020) investigated on vascular risk factors, serving as a biomarker, for MCI to AD conversion in subjects having low cerebral small vessel burden. Memory baseline brain (e.g., hippocampus, entorhinal cortex) and CSF biomarkers were also studied by Kung et al. (2020).

Non-linear Gaussian processes were documented by Lin et al. (2020) to model non-linear interactions of biomarkers including demographics, APOE4, CSF, hippocampal volume, and brain age. The proposed method also provided insight into the biomarker interactions personalized for individual patients (Hojjati et al., 2018). This proposed an extreme learning machine-based method to individually grade multimodal data extracted from MRI images, PET, CSF, and gene biomarkers. Pan et al. (2020) applied several CNN models which were subsequently combined *via* ensemble learning for classifying features extracted from MRI images. AD-NET introduced by Gao et al. (2020) transferred age-related surrogate biomarker information in the form of transfer learning to deep learning of sMRI features for alleviation of data insufficiency.

In this study, we introduce algorithmic methods to characterize and quantify the pathological variations in the identification of biomarkers. Furthermore, extracted biomarkers capable of indicating the severity of AD may be fed into the MLP classifier to differentiate the MCI to AD group from the non-converter MCI group. By honing its capacity through the accumulation of additional data, this framework could be further designed as a computer-aided diagnosis system to assist doctors in taking preventive steps to reduce the progression rate of AD.

## Materials and Methods

### Overview of the Proposed Algorithm

**FIGURE 1:**
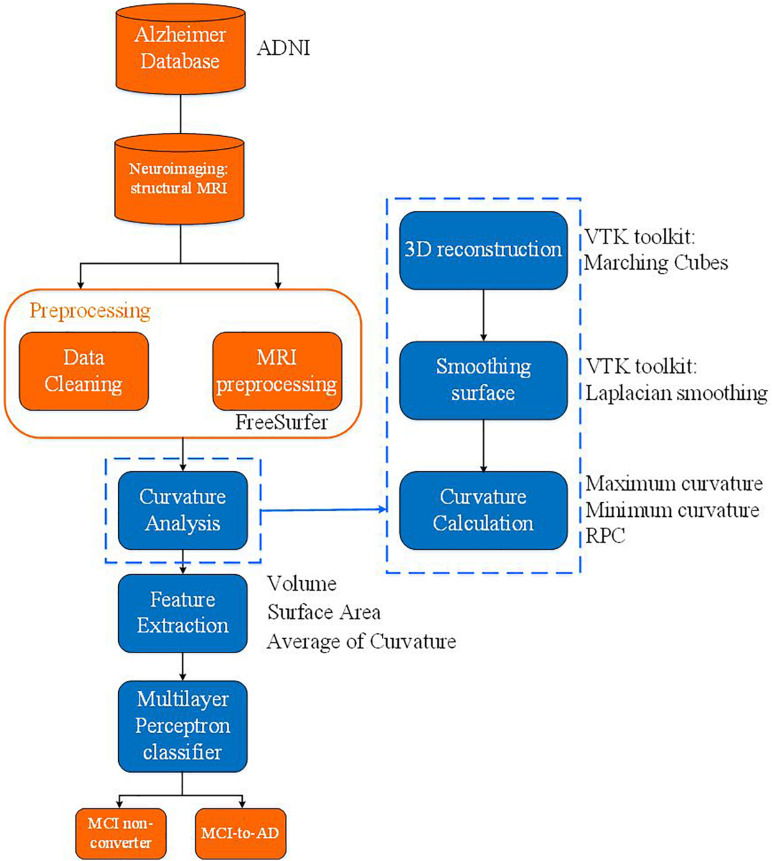
Block diagram of the whole proposed algorithm.

As shown in Figure 1, the proposed algorithm functions in the following order: data pre-processing, feature extraction, and classification after acquiring the MRI images. The overall data pre-processing includes data cleaning for datasets with a series of MRI selection standards and MRI pre-processing and hippocampus segmentation. After obtaining the pre-processed hippocampus-labeled images, the image processing pipeline is followed by a surface construction of the hippocampus. Subsequently, morphological metrics including volume, surface area, and curvature of the hippocampus surface will be calculated. The assessment of the surface curvature will be detailed in the following section since it plays a critical role in our classifier. We perform feature extraction based on a statistical analysis of the volume, surface area, and average RPC, associated to the hippocampal subfields, in the hopes of identifying a proper neural network model to construct the MLP classifier to mimic a neurologist’s decision in assisting with a diagnosis. This MLP classifier serves to identify the converter and non-converter groups.

### MRI Data Acquisition and Selection

MRI data used in this study was obtained from the Alzheimer’s Disease Neuroimaging Initiative (ADNI) database with a primary focus on the analysis of two subject groups, namely, the converter and the non-converter groups. The converters are those cases diagnosed with MCI at the first visit but developing AD within a 2 year period. Their demographic data are shown in Table 1 with the Mini-Mental State Examination (MMSE) scores shown in Table 10. There were no significant differences between the converter and non-converter groups in terms of age at first visit, gender ratio, or years of education as shown in Table 2. The data used for the diagnosis had 1.2 mm resolution and were T1-weighted images acquired from 1.5 to 3.0 T scanners. These data were collected for studies through four phases: ADNI1, ADNI GO, ADNI2, and ADNI3. The current study focuses on demographic, neuropsychological, and structural imaging data from the ADNI GO and ADNI2 phases.

**TABLE 1:** The demographic data for the two study groups.

	**MCI non-converter**	**MCI to AD**
Number of subjects	89	89
Age at first visit (years)	73.4 ± 7.6	74.4 ± 8.0
Males (%)	53 (59.6%)	52 (58.4%)
Years of education	16.3 ± 2.6	15.8 ± 2.6
		

**TABLE 2:** Chi-square test between the MCI non-converter and MCI to AD.

**Type**	**Chi-square**	**Degrees of freedom**	**Significant level 5%**
Gender (M, F)	0	1	3.841
Age (50s, 60s, 70s, 80s, 90s)	8.843	4	9.488
Education (11∼20)	8.905	9	16.919
MMSE (−12, −11, −8∼5)	58.263	15	24.996

Structural MRI images were selected from the ADNI database and were pre-processed with images scanned in the sagittal plane, to minimize differences as a result of individual scanning conditions. Pre-processing includes correcting the non-uniform intensity caused by the gradient warp distortion. The data in the ADNI1 phase was not included in current studies to assure consistent MRI scanning protocol. The ADNI3 phase data was also excluded due to incompleteness. In order to eradicate unexpected factors, a series of data cleaning steps were defined for each cohort as follows. Data from GE were excluded due to difference in protocols in ADNI2 and GO phases: (a) excluded ADNI1 and ADNI3 phases; (b) included pre-processing steps, e.g., MT1, N3, and Gradwrap; (c) excluded the accelerated scanning images; (d) excluded images if the scanning time differs from the diagnosis time by over 2 weeks; (e) chose the scanning images with the scanning time closest to diagnosis time;, and (f) investigated whether the diagnosis record has a missing score in neuropsychological data.

### MRI Pre-processing

Structural MRI pre-processing consists of two parts: one for assuring consistencies due to different scanning systems and the other another for inner-subject variabilities.

The pre-processing for scanning system consistency includes MT1, N3, and Gradwrap. MT1 is a multiplane reconstruction process, in which the scanning image will produce sagittal, coronal, and axial planes. N3 (Sled et al., 1998) is an algorithm for correcting intensity non-uniformity in MRI which is caused by inhomogeneous radiofrequency (RF) excitation. Gradwrap (Axel and Morton, 1989) corrects the gradient distortion which is caused by both gradient non-linearity and imperfections in the B0 field to assure revelation of significant hippocampus features in discerning between the MCI group and the AD group.

Pre-processing for inner-subject data consistency includes a series of step. The first step is size conformation by which image sizes from different manufacturers were normalized. The second step consists of non-uniform intensity normalization which is similar to the pre-processing for assuring scanning system consistency but does not include the magnetic field strength information. The third step is the Talairach transform computation with Talairach coordinates used for brain size and shape normalization. Intersubject registration within the standardized space is used to compare different brain positions with different sizes. The fourth step is interslice intensity normalization which attempts to correct for fluctuations in intensity caused by eddy current and cross talk between slices. This intensity correction step is aimed to enhance the accuracy of the subsequent segmentation process. The fifth step, namely, the skull strip, utilized the watershed algorithm to segment and remove the skull, eyes, and neck which are not part of the brain. The sixth step applies a subcortical segmentation algorithm to segment and label each subcortical structure. It calculates transforms to align the input volume to the Gaussian classifier atlas (GCA). Normalization was also performed using non-linear transforms based on GCA, which labels subcortical structures within the GCA model. The final step is white matter segmentation which uses intensity, neighborhood, and smoothness constraints to segment and separate the white matter.

### Segmentation of the Hippocampus and Its Subfields

After pre-processing, hippocampus segmentation was performed using FreeSurfer 6.0 (Fischl, 2012). This is based on the statistical atlas which was constructed from manual labels and information from Bayesian reference (Iglesias et al., 2015). The FreeSurfer pipeline used in current research generated the masks of the entire hippocampus as well as its subfields according to the pre-trained statistical atlas (Iglesias et al., 2015). The hippocampus was further segmented into 12 subfields, namely, the parasubiculum, presubiculum, subiculum, CA1, CA3, and CA4. Granule cells were in the molecular layer of the dentate gyrus (GC-ML-DG), hippocampus–amygdala–transition area (HATA), fimbria, molecular layer, hippocampal fissure, and hippocampal tail. The parasubiculum, HATA, and fimbria subfields were not included due to low resolution in MRI.

Although there were a few literature documenting quantitative performance evaluations on this automatic subfield segmentation and manual segmentation process, results have shown that the segmentation of subfields using FreeSurfer could achieve high test–retest reliability, with intraclass correlation coefficient (ICC) > 0.9 for most subfields (Whelan et al., 2016). The segmented subfields were also shown to be informative in the analysis of AD (Iglesias et al., 2015). When compared with FIRST, a software which also enables hippocampus segmentation in FSL, FreeSurfer has revealed a higher correlation *via* manual tracing (manual segmentation), with approximately 82 ± 1.5% of the volume overlapping in the left hippocampus and 82 ± 2.8% of the volume overlapping in the right hippocampus (Morey et al., 2009).

There should, in general, not be much difference between 1.5 and 3 T images except possibly minor in the contrast. Recently, Brown et al. (2020) have also documented on test–retest reliability of automated segmentation of the hippocampal subfield procedures *via* T1-weighted images acquired from two models of Siemens scanners.

### Curvature Analysis

**FIGURE 2:**
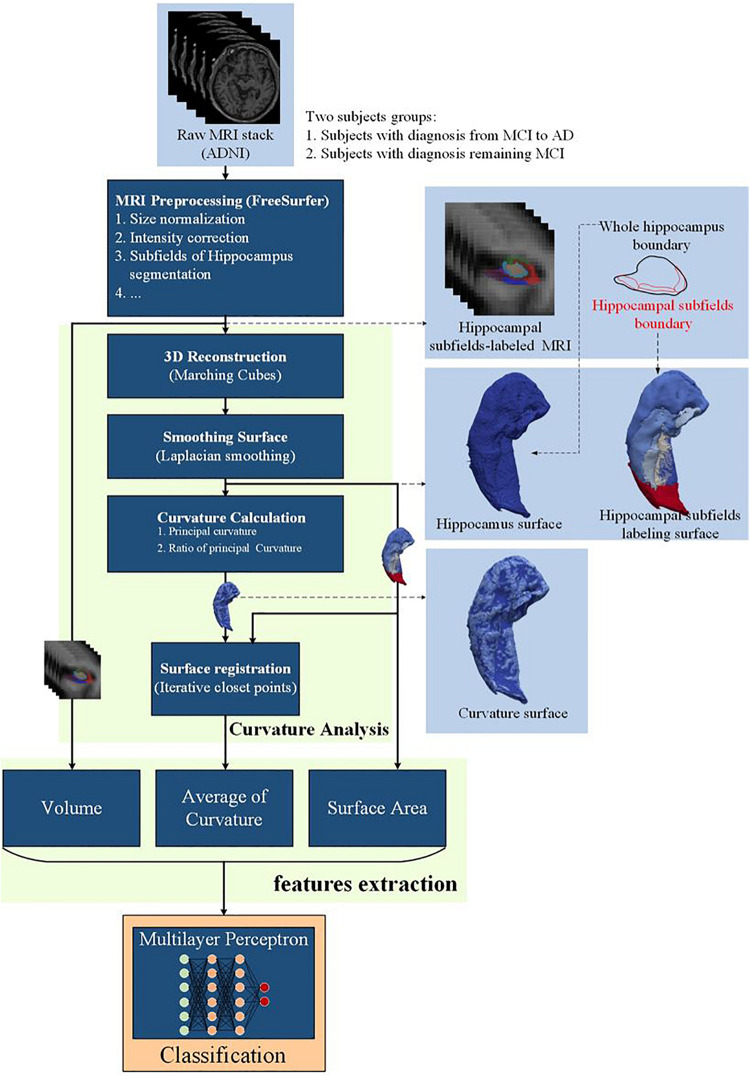
Flowchart of curvature analysis on the hippocampus surface.

**FIGURE 3:**
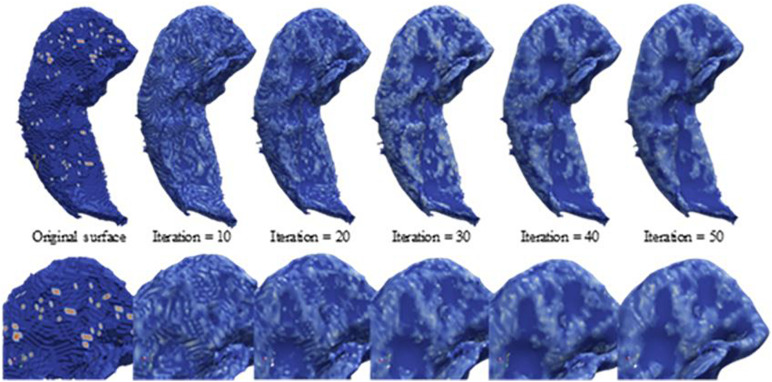
Laplacian smoothing on the hippocampal surface. From left to right, displayed are the series of hippocampal surfaces from the original surface constructed from the Marching cubes to the surface with more smoothing iterations. The upper surfaces are the full size of the hippocampus, and the lower surfaces are about the zooming surface focus on hippocampal head for observing the smoothing levels at a fine scale.

As depicted in Figure 2, the segmented hippocampus and its subfields subsequently underwent 3D surface reconstruction, surface smoothing, and calculation of the curvature indices. The 3D reconstruction is based on the Marching cubes algorithm (Lorensen and Cline, 1987) to produce a surface from hippocampus-labeled MRI. Subsequently, surface smoothing is performed to compensate for the spurious structure of the original segmentation with the Laplacian smoothing algorithm. The effect of surface smoothing is demonstrated in Figure 3. The surface from the original hippocampal segmentation could be quite rough due to limited spatial resolution and SNR in MRI scans. The smoothing algorithm removes the bulgy structure to adequately reveal the surface structure. The number of iterations for smoothing has been set to 50 in this work to balance noise removal and principal feature preservation.

**FIGURE 4:**
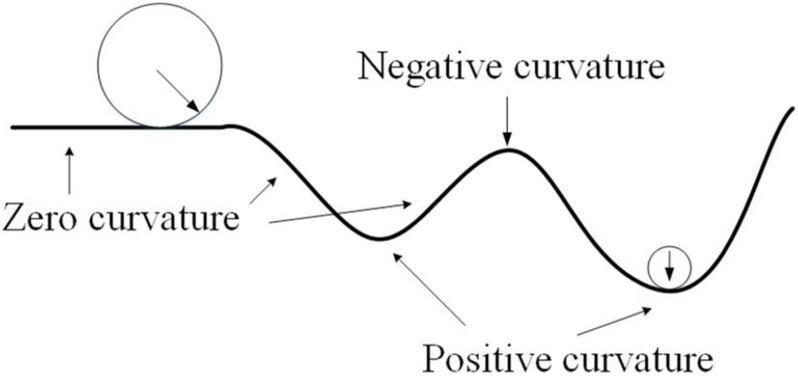
Illustration about the curvature to quantify the degree of bending and folding.

The curvature analysis measures the principal curvatures and RPC. Quantified by the curvature index, the cortical gyrus and sulcus can be described as the juxtaposition of ridges and valleys according to Boucher et al. (2009). Curvature was first defined as the second-order derivative of a 1D curve, as shown in Figure 4. The concept can be generalized to a 2D surface to measure folding conditions in terms of normal curvature, defined by a 1D curve intercepted by a normal plane at a specific point. Among all normal curvatures obtained from the different rotating angles of a given point on the surface, the maximum and minimum are defined as the principal curvatures, respectively. The principal curvatures of a given vertex, *v*, on the surface in 3D, are formulated as follows:

$$c_{m\,a\,x}\,\left(v\right)=H\,\left(v\right)+\sqrt{{\left(H\,\left(v\right)\right)}^{2}-K\,\left(v\right)},\mathrm{and}$$

$$c_{m\,i\,n}\,\left(v\right)=H\,\left(v\right)+\sqrt{{\left(H\,\left(v\right)\right)}^{2}-K\,\left(v\right)}.$$

**FIGURE 5:**
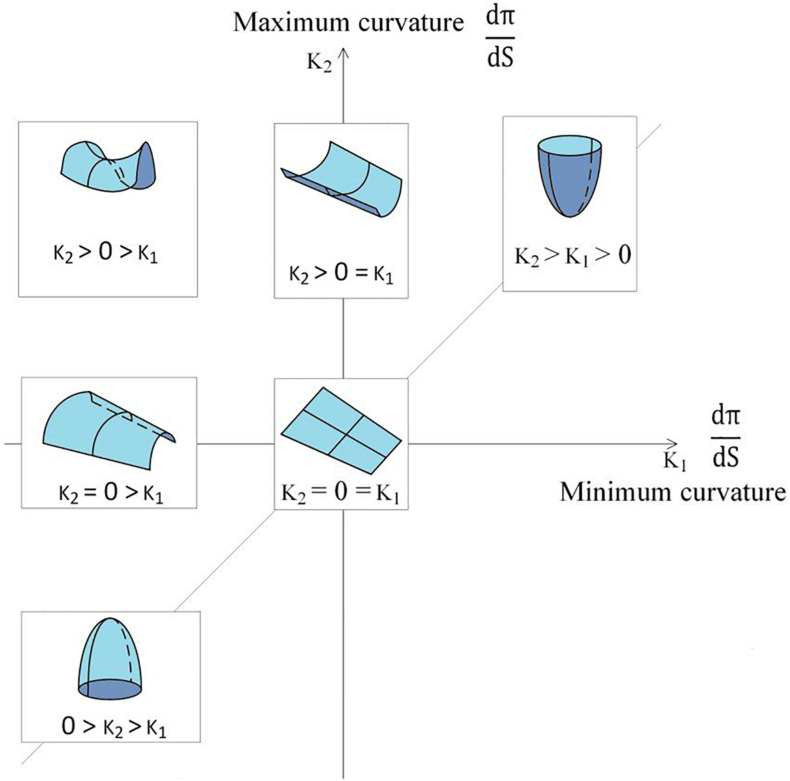
Local folding pattern corresponds to the principal curvature.

The *K*(*v*) indicates the discrete Gaussian curvature at vertex *v* which can be obtained from the Gauss–Bonnet theorem, given by

$$K\,\left(v\right)=d\,\left(v\right)=2\,\pi -\sum_{i}^{N}=0\,\beta_{i},$$

where *N* is the number of faces containing *v* and β_*i*_ is the interior angle of *v*. *H*(*v*) represents the discrete mean curvature defined as

$$H\,\left(e\right)=\frac{1}{N}\,\sum_{i=1}^{N}s\,\left(e\right)\cdot \psi \,\left(e\right)$$

where *e* is the edge set incident to the vertex *v* and *N* is the number of edges in the edge set. Besides, the *s*(*e*) denotes the length of the edge, and _ψ(e)_ denotes the dihedral angle of *e*. Based on the topology observed, we also noted that RPC can be an effective index to distinguish the local folding pattern, as shown in Figure 5, which is defined by

$$R\,P\,C=\frac{M\,a\,x\,\left\{\left|C_{M\,a\,x}\right|,\left|C_{M\,i\,n}\right|\right\}}{M\,i\,n\,\left\{\left|C_{M\,a\,x}\right|,\left|C_{M\,i\,n}\right|\right\}}$$

The curvature indices of the entire hippocampus as well as all the segmented subfields are all calculated. Separation of the curvature of the entire hippocampus and its subfield will help to reveal structural alterations inside the hippocampus during the conversion. Instead of mapping the calculated curvature on the lattice grid of the segmentation map, an iterative closet points (ICP) surface registration algorithm (Besl and Mckay, 1992) has been used to register the curvature values of the subfield segmentation after smoothing. The refined registration was applied out of concern that the curvature of the subfield is prone to a low signal-to-noise ratio and limited spatial resolution.

### Feature Selection for Classification

Proper selection of features as prediction variables has been known to be a critical factor in constructing classification models. In our current work, neuropathological and morphological changes as described by medical experts have been used to characterize the volume, surface area, averages of the principal curvatures, and the average of the RPC biomarkers. These indices featuring the entire hippocampus and its subfields were chosen for investigation while anticipating biomarker identification to categorize the converter and the non-converter cohorts. Furthermore, this work seeks to track symptom changes over time. The change in rate of each morphological index should be more useful than the indices at each time point. Thus, the change in rate (*C*_*f*_) of a morphological index is defined as

$$C_{f}=\frac{f_{s\,e\,c\,o\,n\,d}-f_{f\,i\,r\,s\,t}}{f_{s\,e\,c\,o\,n\,d}},f\in F=\left\{v\,o\,l\,u\,m\,e,s\,u\,r\,f\,a\,c\,e\,\_\,a\,r\,e\,a,a\,v\,e\,r\,a\,g\,e\,\_\,c\,u\,r\,v\,a\,t\,u\,r\,e\right\}$$

to quantify the relative difference between two visits. In the feature selection step, we explore neuroimaging features. Feature selection is carried out because that multilayer perceptron will usually treat all features as equivalent or begin. With the training process, a multilayer perceptron will determine the most suitable parameters in the feature map. As such, reducing some features such as noise can more accurately and quickly yield a suitable weight in the feature map. In the first selection method, the univariate selection, a two-sample *t*-test is conducted to compare the *C_f* in each volume of interest to explore potential useful biomarkers. The indices showing significant differences between the two groups will be adopted as the candidate prediction variables in the classifier. Features are selected on the basis of their *p*-value in an independent *t*-test. The *p*-value may represent whether the difference is sufficiently large to justify the conclusion that the two samples were drawn from different populations. The second method is feature importance, which is based on the features set combinability and Gini impurity, whereby features are selected based on their combination relevance.

### Multilayer Perceptron Classifier

MLP is a popular and efficient neural network model in the field of pattern recognition. MLP mimics the developing brain, the plasticity, and the storage of experiential knowledge, which is also known as learning processing. MLP is a multilayer network capable of multilevel information extraction through its hierarchical structure. MLP may also be seen as a multivariate probabilistic function or mapping of input features to outputs.

**FIGURE 6:**
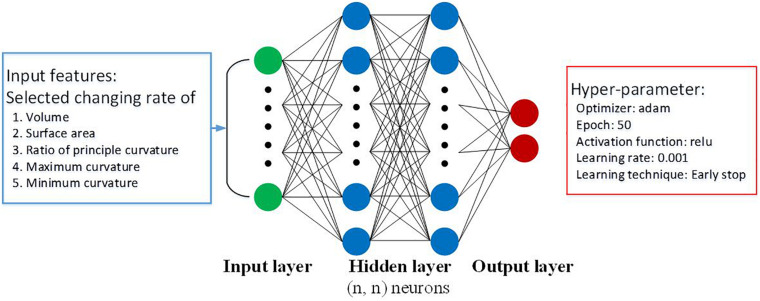
Input features and proposed architecture in MLP.

The MLP architectures proposed in this current research are shown in Figure 6. There are three main parameters needed for the design: (1) the number of hidden layers, (2) the number of neurons in each layer, and (3) the activation function. With regard to choosing the number of hidden layers, we have a shallow design network. Our input features are highly representative, and we do not need a deep network to identify the complicated relations. With regard to choosing the number of neurons in each layer, we used a grid search method to find out the best parameter. In addition, we used ReLU as an activation function. ReLU reduced the probability of a vanishing gradient and is more computationally efficient. According to the design, the MLP is equipped with two hidden layers within each 12-neuron layer.

The prediction was based on these architectures and was performed using the scikit-learn framework. The ReLU function was used as the activation function. The Adam with momentum (0.9) and adaptive learning rate to enhance training was used to optimize the learning process. An L2 norm penalty of weight 0.0001 was also imposed for regularization. The maximum iteration was set to 800. All the subject data were randomly shuffled; 60% of them were treated as the training set, 30% of them were treated as the testing set, and 10% of them were used for validation.

## Results

### Feature Extraction From the Hippocampus

**FIGURE 7:**
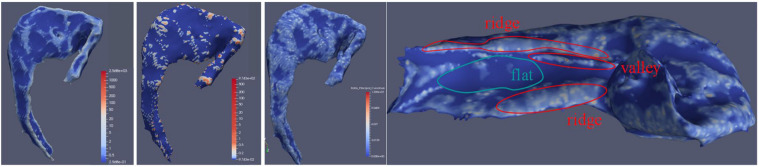
CA1 subfield surface attached with calculated maximum curvature, minimum curvature, and RPC. The leftmost artwork is about the CA1 subfield surface with maximum curvature. The second artwork from the left is about the CA1 subfield surface with minimum curvature. The third artwork from the left is about the CA1 subfield surface with RPC. The right artwork is about the demonstration of RPC by the medial side of the hippocampus surface with RPC attached. In this illustration, the RPC showed its ability to indicate valleys and ridges on the surface.

**FIGURE 8:**
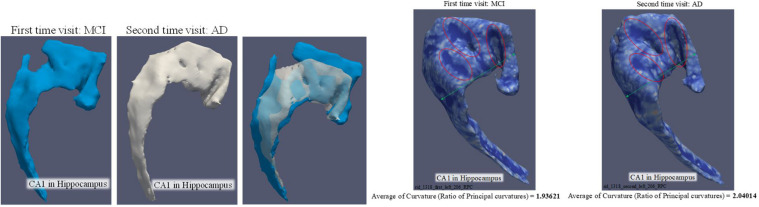
Comparison of CA1 surface between MCI and AD. The three artworks in the left are the comparison of volume and surface area. The volume is 443.964 mm^3^ and surface area is 753.45 mm^2^ when the subject was diagnosed as MCI. The volume is 325.024 mm^3^ and surface area is 594.504 mm^2^ when the subject was diagnosed as AD. The two artworks in the right are targeted to demonstrate the change of “average of RPC.”

The 3D curvature mappings are shown in Figure 7. The maximum curvature is capable of delineating the ridges of the local structure (Figure 7), while the minimum curvature is capable of outlining the cap-shape patterns (Figure 7). While the two principal curvatures enhance the folding regions bending in different directions, the RPC helps to distinguish the folding area and the flatting area. However, the RPC is instrumental in enhancing the morphological complexity of cortical surfaces, as shown in Figure 7, indicating capture of the structural changes in the hippocampus. Figure 8 illustrates the morphological changes in CA1 from a typical converter. Not only the volume but also the surface area was significantly reduced. A smaller flat region was also noticed when the subject was diagnosed with AD with increased RPC.

The rates of change in volume, surface, and RPC data of all nine subfields are listed in Tables 3–5. The converter generally shows a higher reduction level over time in terms of volume and surface area than the non-converter. Most of the subfields reveal statistically significant differences, except for the parasubiculum, HATA, and fimbria. The hippocampus subregion segmentation algorithm will make an error in a small region appear particularly significant since our surface model is constructed by labeled segmentation of MRI data. These errors will continue to be passed on to the feature *via* the feature extraction step. On the other hand, only temporal changes in the average RPC in CA1 achieved significant difference levels between the two groups and reached a statistical significance level quite early in the subiculum. Table 6 shows the selection results for neuroimaging features. For neuroimaging features, we found that volume and surface area are strong features in different subregions. There are only seven curvature features selected by the criteria. We also observed that the presubiculum might be a region of interest since the volume, surface area, RPC, minimum curvature, and maximum curvature have been selected. In addition, the subiculum, CA1, CA4, and the molecular layer have four features which have been selected by a univariate selection method. They might also be regions of interest to carefully focus on. We built 100 random forest classifiers with 20 decision trees. Due to the randomness of the subset of features in forming the decision tree, the model may yield important differences in feature weights each time. By training the model multiple times, for a certain number of cycles, we were finally able to obtain a certain amount of features that make an important contribution to the impact of the classification task. The importance of a feature is computed as the total Gini impurity reduction of the criterion brought by that feature. It is also called the Gini importance. We sort the rank from the whole feature according to the sum of the feature importance with different weights. A higher feature importance rank in the random forest has a higher weight. The order of ranking is representative of the relative importance in Table 7.

**TABLE 3:** Two-sample t-test on changing rate of volume in different hippocampal subfields.

**Regions**	**Changing rate of volume**		***p*-value**	**Cohen’s *d***
	**MCI non-converter group**	**MCI to AD group**		
Presubiculum	−2.73 ± 8.38%	−7.34 ± 6.46%	0.00001	0.61848
Subiculum	−3.96 ± 6.13%	−7.78 ± 5.81%	0.00001	0.64324
CA1	−2.74 ± 5.14%	−4.84 ± 4.99%	0.00154	0.41515
CA3	−3.21 ± 7.99%	−6.13 ± 6.68%	0.00233	0.39884
CA4	−3.07 ± 6.02%	−5.34 ± 5.01%	0.00167	0.41205
GC-ML-DG	−3.52 ± 6.07%	−5.61 ± 4.86%	0.00365	0.38056
Molecular layer	−3.6 ± 6.23%	−6.62 ± 4.71%	0.00003	0.54958
Hippocampal fissure	0.57 ± 7.83%	−3.34 ± 8.93%	0.00037	0.46771
Hippocampal tail	−2.26 ± 8.36%	−4.87 ± 6.3%	0.00681	0.35392

**TABLE 4:** Two-sample t-test on changing rate of surface area in different hippocampal subfields.

**Subfields**	**Changing rate of the surface area**		***p*-value**	**Cohen’s *d***
	**MCI non-converter group**	**MCI to AD group**		
Presubiculum	−2.23 ± 4.95%	−4.75 ± 4.62%	0.00006	0.52750
Subiculum	−2.59 ± 4.14%	−5.68 ± 4.16%	0.00001	0.74995
CA1	−2.36 ± 4.58%	−4.36 ± 3.93%	0.00003	0.47154
CA3	−3.22 ± 5.58%	−5.62 ± 5.29%	0.00075	0.44230
CA4	−2.91 ± 4.39%	−5.01 ± 3.89%	0.00045	0.50678
GC-ML-DG	−3.36 ± 4.87%	−4.87 ± 4.23%	0.00109	0.33237
Molecular layer	−3.25 ± 5.38%	−5.71 ± 4.54%	0.00017	0.49474
Hippocampal fissure	2.53 ± 13.24%	−3.96 ± 13.42%	0.000205	0.48880
Hippocampal tail	−1.61 ± 4.08%	−4.06 ± 4.43%	0.00001	0.57654
				

**TABLE 5:** Two-sample t-test on changing rate of “average of RPC” in different hippocampal subfields.

**Subfields**	**Changing rate of average of RPC**		***p*-value**	**Cohen’s *d***
	**MCI non-converter group**	**MCI to AD group**		
Presubiculum	3.26 ± 7.82%	1.24 ± 9.65%	0.07571	0.23127
Subiculum	−0.24 ± 7.74%	1.87 ± 8.92%	0.05146	0.25377
CA1	0.17 ± 7.1%	2.59 ± 8.33%	0.01595	0.31467
CA3	4.23 ± 14.62%	3.61 ± 14.42%	0.74307	0.04254
CA4	13.0 ± 54.5%	14.3 ± 58.17%	0.85797	0.02323
GC-ML-DG	3.5 ± 20.75%	4.35 ± 19.91%	0.74702	0.04187
Molecular layer	4.08 ± 23.04%	7.0 ± 24.87%	0.34714	0.12212
Hippocampal fissure	2.14 ± 19.4%	2.85 ± 19.14%	0.77527	0.03705
Hippocampal tail	2.12 ± 11.1%	2.11 ± 12.34%	0.99021	0.00159

**TABLE 6:** Selection result based on univariate selection.

**Rank**	**Region**	**Feature**	***p-*value**
1	Subiculum	Surface area	< 0.00001
2	Subiculum	Volume	< 0.00001
3	Presubiculum	Volume	< 0.00001
4	Hippocampal tail	Surface area	0.00001
5	Molecular layer	Volume	0.00003
6	Presubiculum	Surface area	0.00006
7	CA4	Surface area	0.00012
8	Molecular layer	Surface area	0.00017
9	Hippocampal fissure	Surface area	0.00021
10	CA1	Surface area	0.00034
11	Hippocampal fissure	Volume	0.00038
12	CA3	Surface area	0.00076
13	CA1	Volume	0.00155
14	CA4	Volume	0.00168
15	CA3	Volume	0.00234
16	GC-ML-DG	Volume	0.00366
17	Presubiculum	CurvMin	0.00373
18	Hippocampal tail	Volume	0.00681
19	CA1	CurvMax	0.00969
20	GC-ML-DG	Surface area	0.01097
21	CA1	RPC	0.01596
22	Subiculum	RPC	0.05146
23	Presubiculum	CurvMax	0.06049
24	CA4	CurvMin	0.06766
25	Presubiculum	RPC	0.07571
26	Molecular layer	CurvMax	0.11261
27	Hippocampal tail	CurvMax	0.25083
28	Hippocampal fissure	CurvMax	0.29224
29	Molecular layer	RPC	0.34715
30	CA4	CurvMax	0.41302

**TABLE 7:** Selection result based on feature importance.

**Rank**	**Region**	**Feature**	**Score**
1	Presubiculum	Volume	2,335
2	Hippocampal tail	Surface area	2,004
3	Subiculum	Surface area	1,994
4	Presubiculum	Surface area	1,972
5	Hippocampal fissure	Surface area	1,687
6	Subiculum	Volume	1,598
7	Hippocampal fissure	Volume	1,551
8	CA4	Volume	1,545
9	GC-ML-DG	Surface area	1,420
10	GC-ML-DG	Volume	1,374
11	CA4	Surface area	1,349
12	Hippocampal tail	Volume	1,277
13	Presubiculum	CurvMin	1,107
14	Molecular layer	Surface area	1,053
15	Molecular layer	Volume	1,016
16	Molecular layer	CurvMax	694
17	CA3	Volume	663
18	CA1	CurvMax	619
19	Subiculum	CurvMax	612
20	CA4	CurvMin	609
21	Presubiculum	RPC	479
22	CA1	RPC	462
23	CA3	RPC	458
24	Subiculum	RPC	320
25	CA4	CurvMax	306

### Identification of MCI Converting Based on MLP

Performances of the MLP, adopting different combinations of prediction variables, are listed in Table 8. We found the criteria (*p* < 0.01) with a higher accuracy (79.07%) in a univariate selection method. Performance decreased with this criteria (*p* < 0.05; *p* < 0.1). This means that the extra selected features represent noise in the classification task. The second observation was that the criteria (random forest top 20) had a higher accuracy (79.95%) in the feature importance method. The best accuracy was also found in the two-selection method. This is reasonable, since the random forest considered both the combination of feature set and classification ability at the same time. We roughly assessed the different criteria. We will compare the selected features by a two-selection method and remove the common parts. This can help with the interpretation. Using both selection methods can enhance the model accuracy by about 15%.

**TABLE 8:** Performance of the MLP classifier with different input features (100 times training result averaging).

**Input feature**			**Neuroimaging features**		
**Basic MLP architecture**			**2 hidden layers with 12 neurons**		
**Selection method**	**Criteria**	**Number of features**	**Accuracy (%)**	**Sensitivity (%)**	**Specificity (%)**
Univariate selection	*p*-value < 0.01	19	79.07	72.41	85.74
Univariate selection	*p*-value < 0.05	21	78.47	72.04	84.91
Univariate selection	*p*-value < 0.1	25	75.96	71.39	80.00
Feature importance	Random forest top 10	10	72.45	60.00	84.91
Feature importance	Random forest top 15	15	78.10	70.28	85.93
Feature importance	Random forest top 20	20	79.95	74.44	85.46
Feature importance	Random forest top 25	25	76.39	73.52	79.26
Whole feature (without feature selection)		45	65.97	60.56	71.39

### Correlation Between MMSE and Neuroimage Biomarkers

The correlation between the neuroimaging biomarkers with MMSE scores is shown in Table 10. It is observed that based on *p* < 0.05, the volume and surface area markers have revealed high correlations with the presubiculum, subiculum, molecular layer, and hippocampal fissure subfields. In addition, the newly introduced RPC biomarker, representing degeneration, has statistical significance between MMSE at the CA1 and molecular layer subfields. High correlations could also be observed between pathological indicative maximum curvature and minimum curvature biomarkers and MMSE primarily at the CA1 subfield.

## Discussion

**FIGURE 9:**
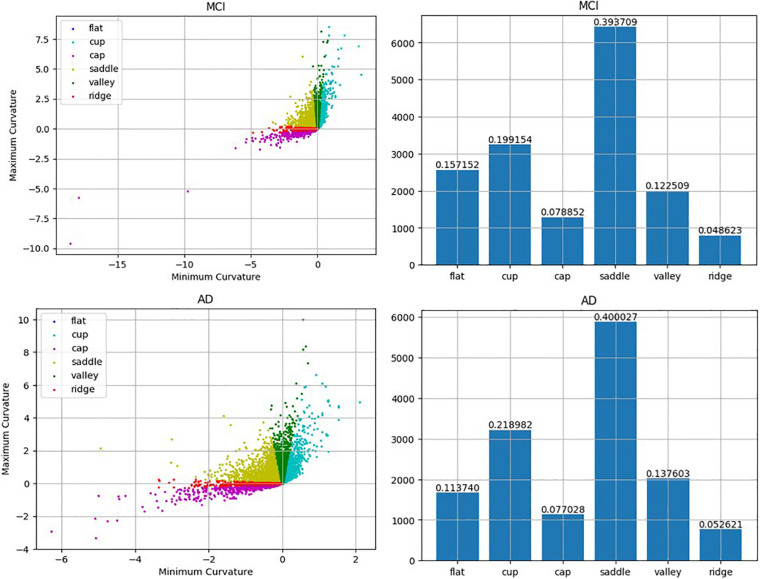
Comparison between MCI and AD according to the detected local folding pattern on the whole hippocampus surface. The two left artworks are the local folding pattern identification of the surface in MCI stage and the surface in AD stage, respectively. Moreover, the two right artworks are the histogram about the proportion ratio of each local folding patterns in the MCI stage and AD stage, respectively.

Previous studies have found that morphological changes in the hippocampus are highly related to the progression of AD. While most research studies have investigated the entire hippocampus as a single unit, the present work has tried to associate the alteration of the subfields of the hippocampus with the progression of cognitive impairment. The results suggested that the rate of change of the average RPC can be used as a biomarker of whether an MCI patient will convert to AD. While the average RPC captures the structural features in AD progression, the ways in which the pattern changes were also qualitatively analyzed. Based on the correspondence between principal curvature indices and morphology, the histogram showing the distribution of the surface pattern from the data of a typical converter subject is plotted in Figure 9. The flat pattern was found to diminish more than the other patterns. This finding suggests that the boundaries of the hippocampal subfields would grow increasingly spurious during conversion to AD. Consequently, RPC has the potential to detect whether a subject has developed AD according to the change in surface pattern.

Several studies have focused on the link between volume and surface area while discriminating MCI and AD. These two structural quantities have shown their potential in the classification. Consequently, this work has invested much effort into investigating these two quantities and their temporal changes, as shown in Tables 3, 4. The experimental results also showed that the curvature features can improve the classification ability. Curvature features which have statistical meaning and important features have been selected by univariate selection. As a result, the combination of the random forest top 20 features is likely to provide the highest accuracy in predicting changes in symptoms based on the presented framework. However, there is still room to improve the prediction accuracy, and we anticipate that further factors, regardless of morphology or physiology, should be further investigated to increase the quality of the classification.

The architecture of the MLP classifier is also key to the prediction accuracy, in addition to properly selecting the prediction features. After obtaining the optimized prediction results for the two hidden-layer MLP, we tried modifying the MLP to a larger number of layers and neurons. However, as far as the number of subjects is concerned, the MLP structure cannot go too much deeper lest overfitting takes place. Another interesting fact in the experiment is shown in Table 8, whereby the increase in input features does not guarantee a better prediction, even when combining the three prominent features. These two findings suggest that the design of an optimal MLP classifier requires sophisticated tests. While there is no standard way to optimize the variables and parameters, our approach, starting with a statistical analysis on the feature selection, may greatly ease the daunting procedure of optimizing the structure of the classifier.

Several studies have sought to discriminate between MCI converters (MCI-C) and MCI non-converters (MCI-NC). A list of the accuracy from the literature is listed in Table 9. This study achieved a 79.95% accuracy based on the MLP classifier with a surface area and average RPC as input features, while the accuracy of the other approaches ranged from 74.1 to 83.3%. We have found that the present work has achieved relatively high accuracy, with only MRI data extracted from the regions in the hippocampus without combining other features from PET, CSF, or cognitive scores. This work could be used as a good screening tool for clinical examinations, as only a structural MRI scan is required with a simple MLP classifier. The loads for scan and computation are minimal. Given that other examination data may provide complimentary information to improve prediction quality, the MLP classifier can be augmented to accommodate more information to ensure better accuracy when a MCI converter is suspected.

**TABLE 9:** Classification comparison of different approaches (order follows the time of publication).

**Study**	**ROI**	**Subjects**	**Features**	**Accuracy (%)**
Chupin et al. (2009)	Hippocampus and amygdala	76 MCI-C 134 MCI-NC	MRI (volume)	67.0
Misra et al. (2009)	Whole brain	27 MCI-C 76 MCI-NC	MRI (RAVENS score)	75–80
Liu et al. (2010)	Hippocampus, amygdala, and caudate	21 MCI-C 79 MCI-NC	MRI (volume)	69.0
Davatzikos et al. (2011)	Whole brain	69 MCI-C 170 MCI-NC	MRI (SPARE-AD) + CSF	61.7
Wolz et al. (2011)	Whole brain	167 MCI-C 238 MCI-NC	MRI (hippocampus volume)	65.0
			MRI (hippocampus volume, thickness, TBM, and manifold-based learning)	68.0
Duchesne and Mouiha (2011)	Whole brain	20 MCI-C 29 MCI-NC	MRI	72.3
Costafreda et al. (2011)	Hippocampus	22 MCI-C 81 MCI-NC	MRI (shape), cognitive scores (MMSE)	80.0
Coupe et al. (2012)	Entorhinal cortex and hippocampus	167 MCI-C 238 MCI-NC	MRI (volume, SNIPE)	73.0
Cheng et al. (2015b)	GM	43 MCI-C 56 MCI-NC	MRI (volume) + CSF + PET	70.7
Ewers et al. (2012)	Hippocampus	58 MCI-C 72 MCI-NC	MRI (hippocampus volume), CSF P-tau181, Aβ1-42, cognitive scores (TMT-B), age	76.9
Westman et al. (2012)	Whole brain	81 MCI-C 81 MCI-NC	MRI (volume, thickness) + CSF	68.5
Zhang et al. (2012a)	Whole brain	43 MCI-C 48 MCI-NC	MRI (volume) + CSF + PET	73.9
Zhang et al. (2012b)	Whole brain	38 MCI-C 50 MCI-NC	MRI (volume) + PET + cognitive scores (MMSE, ADAS-Cog)	78.4
Young et al. (2013)	Whole brain	47 MCI-C 96 MCI-NC	MRI + CSF + PET + APOE	74.1
Wee et al. (2013)	Whole brain	89 MCI-C 111 MCI-NC	MRI (volume, thickness)	75.05
Suk and Shen (2013)	Whole brain	43 MCI-C 56 MCI-NC	MRI (volume) + CSF + PET + cognitive scores (MMSE, ADAS-Cog)	75.8
Eskildsen et al. (2013)	Whole brain	166 MCI-C 134 MCI-NC	MRI (thickness)	80.9
Suk et al. (2014)	Whole brain	76 MCI-C 128 MCI-NC	MRI + PET	75.9
Liu et al. (2015)	Whole brain	117 MCI-C 117 MCI-NC	MRI	78.9
Cheng et al. (2015a)	GM	43 MCI-C 56 MCI-NC	MRI (volume) + CSF + PET	80.1
Suk et al. (2015)	Whole brain	43 MCI-C 56 MCI-NC	MRI	69.3
			MRI + CSF + PET + cognitive scores (MMSE, ADAS-Cog)	83.3
Cabral et al. (2015)	Whole brain	25 MCI-C 56 MCI-NC	MRI+PET	74.0
Moradi et al. (2015)	GM	164 MCI-C 100 MCI-NC	MRI, age, cognitive scores (MMSE, ADAS-Cog, CDR-SB, RAVLT, FAQ)	82.0
Korolev et al. (2016)	Left hippocampus, middle temporal gyrus, inferior parietal cortex	139 MCI-C 120 MCI-NC	MRI (volume, thickness) + plasma proteomic data + cognitive scores (ADAS, RAVLT, FAQ)	79.9

*ROI, regions of interest; GM, gray matter; TBM, tensor-based morphometry; SNIPE, Scoring by Non-local Image Patch Estimator; CDR-SB, Clinical Dementia Rating-Sum of Boxes; ADAS, Alzheimer’s Disease Assessment Scale; RAVLT, Rey Auditory Verbal Learning Test; FAQ, Functional Activities Questionnaire; MCI-C, MCI converters; MCI-NC, MCI non-converters.*

Normalization is a key step in the FreeSurfer to register a brain to the template for subfield segmentation (Wisse et al., 2020). Despite concerns on the accuracy in volumetry of the automatic segmentation, the results suggest that the two different groups, MCI converter and MCI non-converters, could still be distinguishable statistically following the presented procedure. We believe the outcome can be greatly improved providing more accurate information if the anatomical image at a higher resolution becomes available.

Recent advances in machine learning-based biomarker studies for AD are generally faced with two major challenges: the first on diagnostic confirmation of the disease without biopsy and second on data insufficiency. Statistical models are introduced to model the degenerative process which, however, are independent or irrespective of pathological feedbacks. Supervised deep learning *via* CNN has gained high popularity, and information such as, e.g., clinical data, has been transferred to train CNN using neuroimaging biomarkers. To address both challenges, our main contribution is on the introduction of a machine learning algorithm which incorporates the neuropathologist’s experiences in characterizing pathological morphology of the disease in the form of subfield biomarker, primarily curvature analysis, with features selected *via* univariate *t*-test and random forest.

In Table 10, hippocampal volumetric MRI measurements have revealed statistical significance which is consistent with well-established outcomes in clinical AD progression research. In addition to characterizing the degenerative process *via* coarse or global features using volume and surface area, we have further introduced curvature as fine or local neuropathological features delineated at the hippocampal subfields.

**TABLE 10:** Correlation between MMSE and neuroimage biomarkers.

**Regions**	**Feature**	**MMSE**	
		**Pearson’s *r***	***p*-value**
Presubiculum	Volume	0.3166	<0.0001
	Surface_area	0.3399	<0.0001
	RPC	0.01606	0.8316
	Max_Curvature	0.0268	0.7248
	Min_Curvature	–0.0815	0.2836
Subiculum	Volume	0.3093	<0.0001
	Surface area	0.3352	<0.0001
	RPC	–0.0437	0.5669
	Max_Curvature	–0.0541	0.4782
	Min_Curvature	–0.0666	0.3843
CA1	Volume	0.0974	0.1984
	Surface area	0.1059	0.1618
	RPC	–0.1639	0.0303
	Max_Curvature	–0.2193	0.0034
	Min_Curvature	0.1938	0.01
CA3	Volume	0.09047	0.2325
	Surface area	0.1353	0.0734
	RPC	–0.0394	0.6039
	Max_Curvature	0.00488	0.9488
	Min_Curvature	–0.0581	0.4424
CA4	Volume	0.06013	0.4266
	Surface area	0.1001	0.1848
	RPC	0.0023	0.9763
	Max_Curvature	–0.1453	0.0642
	Min_Curvature	0.07523	0.3339
GC-ML-DG	Volume	0.1068	0.1585
	Surface area	0.08517	0.2597
	RPC	–0.0482	0.5233
	Max_Curvature	0.06343	0.4003
	Min_Curvature	0.03293	0.6662
Molecular layer	Volume	0.2298	0.0022
	Surface area	0.2101	0.0051
	RPC	–0.2311	0.0019
	Max_Curvature	–0.1014	0.1952
	Min_Curvature	0.02585	0.7342
Hippocampal fissure	Volume	0.1829	0.0145
	Surface area	0.2416	0.0012
	RPC	0.04782	0.5262
	Max_Curvature	0.09744	0.1996
	Min_Curvature	0.04733	0.546
Hippocampal tail	Volume	0.09862	0.1928
	Surface area	0.121	0.1097
	RPC	–0.0423	0.577
	Max_Curvature	0.06056	0.4273
	Min_Curvature	–0.0475	0.5294

Based on a recently published paper (Gao et al., 2020), the highest accuracy for MCI to AD conversion using only structural MRI is 73%. The accuracy could reach 79% after adding PET markers. Our proposed technique achieves one of the highest accuracy by using only structural MRI biomarker. Medical experts’ intelligence is subsequently augmented with the semisupervised algorithm, primarily *via* the MLP decision-making process using limited imaging data. These biomarkers are further confirmed from the neuropsychological MMSE information.

## Conclusion

The present work has assessed the structural information of the hippocampal subfields, including volume, surface area, and surface pattern as characterized by a curvature analysis. The combined biomarkers of the rate of change in volume, surface area, and curvature from the hippocampal subregions are considered critical in classifying an MCI converter, which can achieve an accuracy of 79.95%.

## Data Availability Statement

Publicly available datasets were analyzed in this study. This data can be found here: https://ida.loni.usc.edu/login.jsp?project=ADNI.

## Author Contributions

Y-MK and GGCL were responsible for the study concept. T-HK and Y-RX carried out the experimental analysis and drafted the manuscript. T-CC provided technical support in analyzing the data and organizing the draft. All authors discussed the results and contributed to the final manuscript.

## Conflict of Interest

T-HK, Y-RX, and GGCL were employed by company MediaTek Inc., Hsinchu, Taiwan. The remaining authors declare that the research was conducted in the absence of any commercial or financial relationships that could be construed as a potential conflict of interest.
